# Genetic diagnosis of fetal microcephaly at a single tertiary center in China

**DOI:** 10.3389/fgene.2023.1112153

**Published:** 2023-05-09

**Authors:** You Wang, Fang Fu, Tingying Lei, Li Zhen, Qiong Deng, Hang Zhou, Chunling Ma, Ken Cheng, Ruibin Huang, Ru Li, Qiuxia Yu, Lushan Li, Jin Han, Xin Yang, Dongzhi Li, Can Liao

**Affiliations:** ^1^ The First School of Clinical Medicine, Southern Medical University, Guangzhou, China; ^2^ Department of Prenatal Diagnostic Center, Guangzhou Women and Children’s Medical Center, Guangzhou Medical University, Guangzhou, China; ^3^ School of Medicine, South China University of Technology, Guangzhou, China

**Keywords:** fetal microcephaly, chromosomal microarray analysis, prenatal diagnosis, whole exome sequence, genetic counseling

## Abstract

**Background:** Microcephaly is common in patients with neuropsychiatric problems, and it is usually closely related to genetic causes. However, studies on chromosomal abnormalities and single-gene disorders associated with fetal microcephaly are limited.

**Objective:** We investigated the cytogenetic and monogenic risks of fetal microcephaly and evaluated their pregnancy outcomes.

**Methods:** We performed a clinical evaluation, high-resolution chromosomal microarray analysis (CMA), and trio exome sequencing (ES) on 224 fetuses with prenatal microcephaly and closely followed the pregnancy outcome and prognosis.

**Results:** Among 224 cases of prenatal fetal microcephaly, the diagnosis rate was 3.74% (7/187) for CMA and 19.14% (31/162) for trio-ES. Exome sequencing identified 31 pathogenic or likely pathogenic (P/LP) single nucleotide variants (SNVs) in 25 genes associated with fetal structural abnormalities in 37 microcephaly fetuses; 19 (61.29%) of which occurred *de novo*. Variants of unknown significance (VUS) was found in 33/162 (20.3%) fetuses. The gene variant involved included the single gene *MPCH 2* and *MPCH 11*, which is associated with human microcephaly, and *HDAC8, TUBGCP6, NIPBL, FANCI, PDHA1, UBE3A, CASK, TUBB2A, PEX1, PPFIBP1, KNL1, SLC26A4, SKIV2L, COL1A2, EBP, ANKRD11, MYO18B, OSGEP, ZEB2, TRIO, CLCN5, CASK,* and *LAGE3*. The live birth rate of fetal microcephaly in the syndromic microcephaly group was significantly higher than that in the primary microcephaly group [62.9% (117/186) vs 31.56% (12/38), *p* = 0.000].

**Conclusion:** We conducted a prenatal study by conducting CMA and ES for the genetic analysis of fetal microcephaly cases. CMA and ES had a high diagnostic rate for the genetic causes of fetal microcephaly cases. In this study, we also identified 14 novel variants, which expanded the disease spectrum of microcephaly-related genes.

## 1 Introduction

Microcephaly is associated with an etiologically heterogeneous group of conditions and is defined as a head size less than three standard deviations (SD) of the mean head circumference at a specific gestational age (Gelber et al., 2017). The incidence of microcephaly in live births is 1.3–150 per 10,000 (Morris et al., 2016). It is the result of genetic abnormalities in the growth of the cerebral cortex during the proliferation of nerve cells. Under normal conditions, the proliferation of nerve cells reaches its peak at two to 4 months of gestation (Pescia et al., 1983). Microcephaly can have genetic underpinnings which may be characterized by trisomy 13, trisomy 18, trisomy 21, single gene defects, Meckel-Gluber syndrome, Smith-Lemli-Opitz syndrome, Bloom syndrome, Nijmegen rupture syndrome, De Lang syndrome, etc (Nawathe et al., 2018). Non-genetic factors include intrauterine growth restriction, prenatal infection, toxoplasmosis, cytomegalovirus, Zika virus, HIV, prenatal exposure to drugs, fetal alcohol syndrome, fetal incontinence syndrome, congenital metabolic abnormalities, maternal phenylketonuria syndrome, etc. (Nawathe et al., 2018). A cohort study involving 680 cases of fetal microcephaly found that genetic factors accounted for about half of the causes of fetal microcephaly, and perinatal fetal brain injury accounted for about 48% (including fetal intrauterine infection and a history of teratogenic exposure) (von der Hagen et al., 2014). Some studies have shown a correlation between the severity of microcephaly and the severity of intellectual disability (Pryor and Thelander, 1968; Dolk, 1991). Thus, microcephaly places a significant financial burden on healthcare systems and families. Microcephaly can be categorized as syndromic microcephaly or identified as a part of several heterogeneous disorders (i.e., syndromic microcephaly), such as chromosomal disorders. The prenatal diagnosis of microcephaly is extremely difficult, and most cases aren’t diagnosed until mid-to-late pregnancy. Thus, early prenatal genetic testing to identify the cause of microcephaly can help with perinatal diagnosis and prognosis. However, only a few studies have investigated chromosomal abnormalities and single-gene disorders in prenatal microcephaly.

Primary and secondary microcephaly are distinguished by the fact that primary microcephaly is characterized by a relatively static level of intellectual degeneration and brain volume deficit. In contrast, secondary microcephaly is characterized by progressive brain degeneration. Primary microcephaly encompasses non-genetic microcephaly and microcephaly primary hereditary (MCPH). In a genetic study of three Canadian patients with microcephaly, Guernsey et al. eventually demonstrated that the MCPH4 disease was caused by pathogenic variants in the gene, *CEP152* (Guernsey et al., 2010). In 2010, Bilguvar et al. (Bilgüvar et al., 2010) discovered five microcephaly patients with homozygous variants in *WDR62*. Nicholas et al. (2010) discovered the second of seven MCPH families with *WDR62* gene variants.

Many researchers have used genetic technology to detect microcephaly (Tan et al., 2017), and several variants have been found to correlate with microcephaly. A study published in 2017 reported that *OTUD7A*, *BBC3*, *CNTN6*, and *NAA15* were associated with microcephaly, which was detected by CMA (Tolezano et al., 2022). The CMA also showed that the presence of 16p13.11 microdeletion along with *NDE1* variant is associated with severe congenital microcephaly (Tan et al., 2017). ES is also a useful technology in detecting microcephaly-related variants. Entezam et al. found that the *PNKP* gene variant was associated with microcephaly (Entezam et al., 2019). The ES was also used to identify three novel variants in the *ASPM* gene associated with primary microcephaly among Saudi families (Naseer et al., 2020). These results supported that both CMA and ES might be used for diagnosing microcephaly. In this study, we used CMA and ES to perform a comprehensive genetic study. We investigated the genetic etiology of fetal microcephaly and provided additional genetic information for patient prognosis and pregnancy decisions.

## 2 Materials and methods

### 2.1 Patient recruitment

**FIGURE 1:**
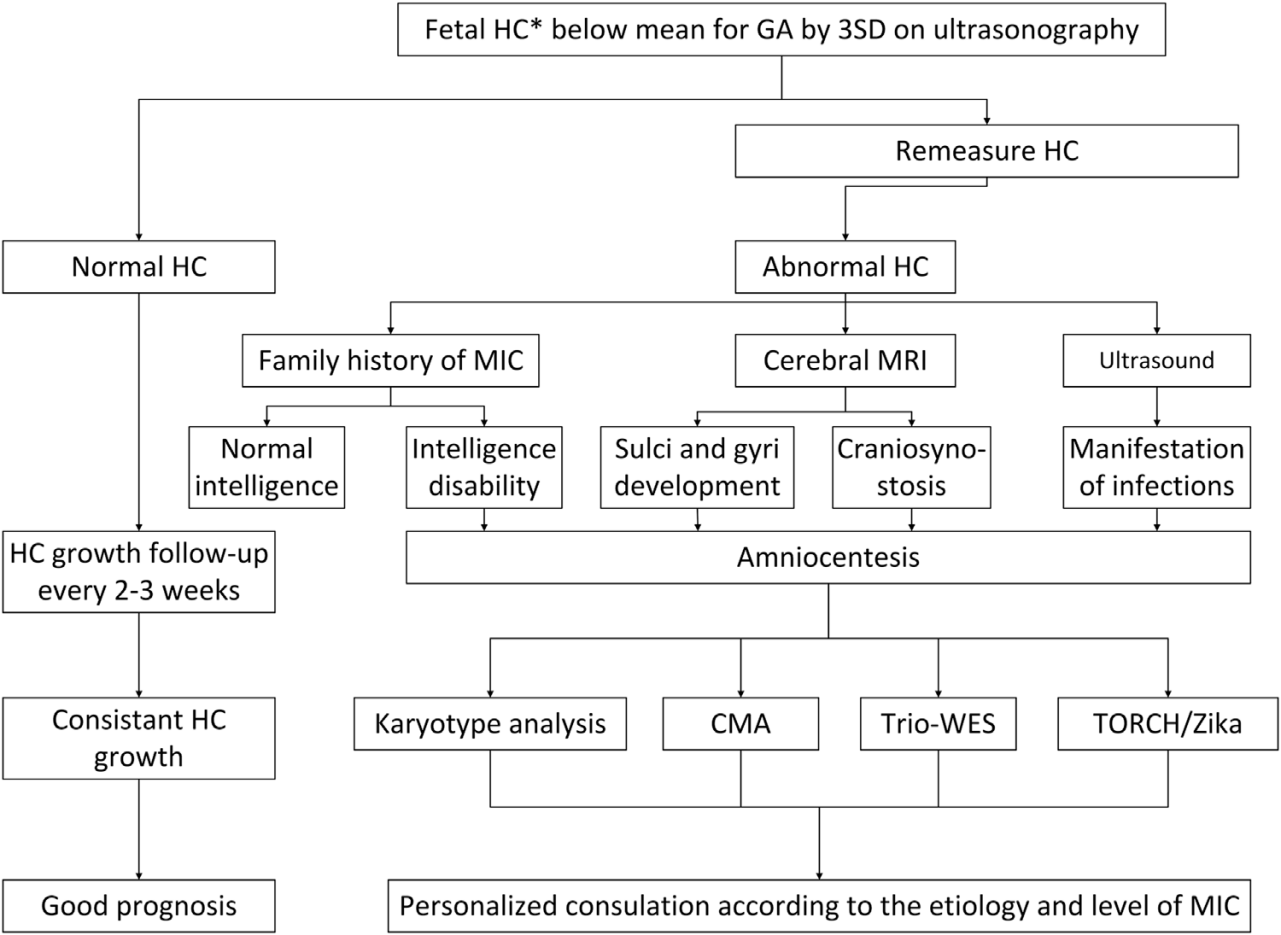
A flowchart for the diagnosis and management of prenatal fetal microcephaly. HC, head circumference; MRI, magnetic resonance imaging; CMA, chromosomal microarray analysis; ES, exome sequencing; MIC, microcephaly; TORCH, toxoplasma, rubella virus, cytomegalovirus, herpes simplex virus, others.

After confirming the gestational week based on the last menstrual period of the patient or early pregnancy ultrasound examinations, 324 parents of fetuses with three SDs of head circumference less than the mean head circumference for gestational age (at 16–37 weeks of gestation) were invited to participate in this study. We followed the latest guidelines of the Canadian Association of Obstetricians and Gynecologists while conducting the study. The flowchart is shown in Figure 1. We selected participants with a singleton pregnancy. Participants received pre-test genetic counseling on the benefits and limitations of CMA and ES for prenatal diagnosis of microcephaly. During invasive prenatal diagnosis, fetal samples were obtained by amniocentesis (<25 weeks) and from umbilical cord blood samples (≥25 weeks or presence of oligohydramnios). Parental blood samples were collected during prenatal diagnosis. In our study, the time from collecting samples to reporting results was 2–12 weeks, and all results were available before delivery. Fetuses with abnormal karyotypes (36 fetuses) were excluded from this study. We collected demographic and genetic data, including maternal age, gestational age at suspected microcephaly, degree of microcephaly, other structural abnormalities associated with microcephaly, chromosomal, and genetic results of invasive surgery, perinatal outcomes, etc. The medical record system was accessed, and telephone follow-ups were performed to collect data on postnatal outcomes. Written informed consent from the parents was obtained and approved by the Institutional Ethics Review Board of Guangzhou Women and Children’s Medical Center.

### 2.2 Sample preparation

Genomic DNA was extracted from 2 mL of cord blood or whole blood or 15 mL of amniotic fluid using the QIAamp DNA Blood Midi/Mini Kit (Qiagen GmbH), following the manufacturer’s instructions.

### 2.3 Chromosomal microarray analysis

A Qiagen DNA Blood Midi/Mini Kit was used to extract fetal DNA from amniocytes and umbilical blood (Qiagen GmbH, Hilden, Germany). A Multiplex Ligation-Dependent Probe Amplification (MLPA) Kit was used to analyze invasive samples by performing quantitative fluorescent polymerase chain reaction (QF-PCR) to detect aneuploidy for chromosomes 13, 18, 21, X, and Y and verify or rule out maternal cell contamination (Guangzhou Darui Biotechnology Co., Ltd., Guangdong, China). Thus, QF-PCR was performed to exclude aneuploidy, and CMA was only performed on samples when the results of the QF-PCR were normal. CMA was performed using a suitable kit (Affymetrix Inc., Santa Clara, CA, United States) following the manufacturer’s instructions. The single-nucleotide polymorphism (SNP) arrays and an array-based comparative genomic hybridization (aCGH) platform on the Affymetrix CytoScan HD/750K arrays had resolutions of 10 and 100 kb, respectively. The results from both CMAs were reported using the International Human Cytogenomic Nomenclature System (ISCN 2020). After generating the GRCh37/hg19 genome, specific genomic regions were assessed. The public databases interrogated in the process of variant classification included ClinGen resource (https://www.clinicalgenome.org/), ClinVar (https://www.ncbi.nlm.nih.gov/clinvar/), University of California Santa Cruz (UCSC, http://genome.ucsc.edu/hg19), OMIM (https://www.omim.org), DECIPHER (http://decipher.sanger.ac.uk/), and ISCA (https://www.Iscaconsortium.org/).

### 2.4 Exome sequencing analysis

If the QF-PCR and CMA results were normal or any non-diagnostic CMA abnormality, the samples were analyzed via trio-ES. ES includes whole exome sequencing and clinical exome sequencing. The DNA samples were enriched following the manufacturer’s instructions (V6, Life Technologies, United States) using Agilent SureSelect Human Exome Capture probes. Pair-end 150-bp reads from Hiseq XTen (Illumina, Inc., San Diego, CA, United States) were applied to the sequence libraries. At a 20x depth threshold, the samples showed greater than 99% coverage. Variants were identified using the NextGENe^®^ v2.4.1.2 software (SoftGenetics, State College, Pennsylvania). All selected variants were divided into five groups, including pathogenic (P), likely pathogenic (LP), VUS, potentially benign, and benign, following the joint consensus recommendations of the American College of Medical Genetics (ACMG) and Clinical Genome Resource (ClinGen) (Riggs et al., 2020). However, this study did not consider benign and potentially benign variants. We applied clinical significance to variants in ClinVar that received three or four stars (i.e., those reviewed by an expert panel or society). The classification also considered the literature on disease relevance, family segregation, *in silico* prediction, maximum minor allele frequency, and the ClinGen expert panel. After filtering out the synonymous and common SNPs (MAF >0.1%), rare variants with high confidence were considered disease-causing candidates. Detailed information regarding the analysis and interpretation of the ES data is provided in Supplementary Material S1.

**FIGURE 2:**
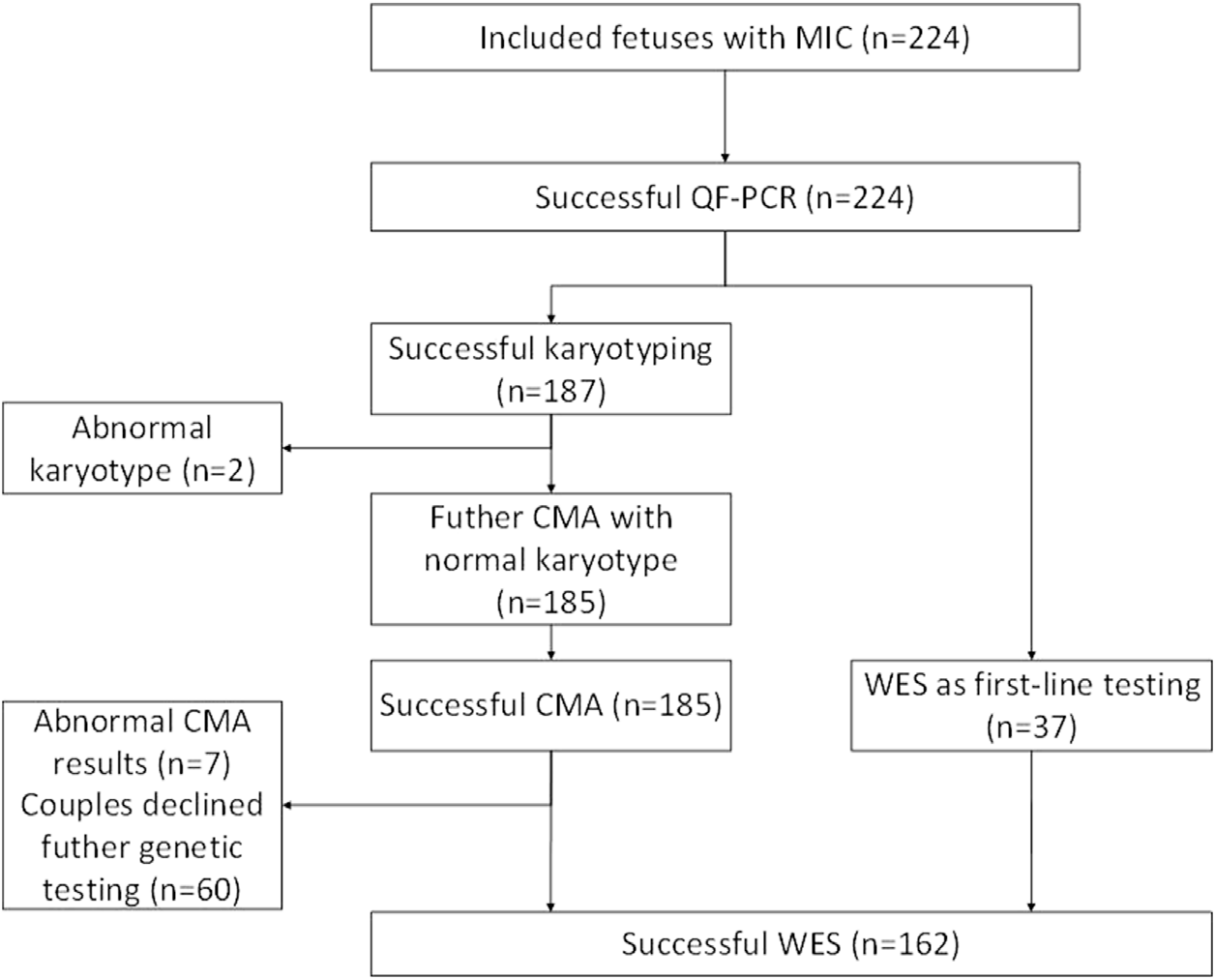
A flowchart for the genetic analysis of a cohort of fetuses with microcephaly. MIC, microcephaly; CMA, chromosomal microarray analysis; ES, exome sequencing; QF-PCR, quantitative fluorescent polymerase chain reacti.

A team consisting of clinical and molecular geneticists, genetic counselors, neonatologists, physicians specializing in maternal-fetal medicine, bioinformaticians, and imaging specialists reviewed and categorized all the qualifying variants to ascertain their relevance to the clinical presentation. We analyzed all reported copy-number variants to ensure that the classification was up to date with the information in public databases and was based on previous studies. Various open databases have been used to categorize copy-number variants, including ClinGen resource, the Genomic Variant Database, ClinVar, and the University of California Santa Cruz (UCSC). Sanger sequencing was performed to confirm variations considered clinically significant (Zhou et al., 2022). Our study included 224 cases, of which 187 cases were analyzed by CMA, 162 cases were analyzed by ES, and 37 cases were analyzed by trio-ES without CMA (These couples rejected the CMA). The average depth of the target area of ES was above 100X, and the coverage of the target area was above 99.75%. Amniocentesis was performed in 149 cases (66.52%), and percutaneous umbilical blood collection was performed in 75 cases (33.48%). The flowchart is shown in Figure 2.

### 2.5 Intelligence, vision, and growth assessment

Neurodevelopmental evaluations included the assessment of physical, motor, sensory, and cognitive development in infants and young children and the measurement of intelligence quotient or adaptive level of functioning among older children. The neurodevelopmental assessment was performed as described in previous studies (Lanzieri et al., 2017; Tann et al., 2018). Intellectual disability evaluation was done using one of the following tests: 1) Bayley Scales of Infant Development; 2) Wechsler Intelligence Scale for Children—third edition; 3) McCarthy Scales of Children’s Abilities; 4) Wechsler Abbreviated Scale of Intelligence; 5) Kaufman Assessment Battery for Children; 6) Stanford Binet Intelligence Scale2; 7) Leiter International Performance Scale–Revised. The scores of all tests were standardized to an average of 100, with a standard deviation of 15 or 16, which allowed us to combine the scores of patients of different ages using different instruments. For patients who received the Bayley Scales of Infant Development at the last assessment when they were ≥42 months old, we calculated the intelligence quotient based on the age-equivalent score divided by the age at the time of the test multiplied by 100. When the standard scores of the latest assessment were <70, 70–84, and ≥85, we divided the patients into intellectual disability, borderline intelligence, and normal intelligence (Sampath et al., 2005; Lanzieri et al., 2017).

We used Griffiths Mental Developmental Scales-II (GMDS) to evaluate an overall Development Quotient (DQ) from the six subscales (A to F). Trained staff who were certified in GMDS and blind to the presence of conditions, clinical history, and imaging results assessed the data.

The ophthalmologic evaluation included external ocular examination, indirect ophthalmoscopy, and measurement of the best corrected visual acuity (Snellen score) in subjects. We defined an ophthalmic abnormality as any of the following conditions: chorioretinitis, optic nerve atrophy, strabismus (including esotropia or exotropia), nystagmus, amblyopia, or astigmatism. Based on the last ophthalmologic visit, we categorized cases/patients as having normal vision (≤20), mild/moderate vision impairment (21–69), low vision (70–199), and legal blindness (≥200), using the Snellen scores for the better eye or based on the assessment of the ophthalmologist for nonverbal and pre-verbal children (Rice et al., 2004).

### 2.6 Statistical analysis

The IBM Statistical Program SPSS 26.0 was used to conduct all statistical analyses. All qualitative data were expressed as n (%). Chi-squared tests were conducted to evaluate count data. All differences were considered to be statistically significant at *p* < 0.05.

## 3 Results

### 3.1 Clinical data of the participants

This was a study of cases of fetal microcephaly at Guangzhou Women and Children’s Medical Center recorded from January 2012 to December 2021. We screened 324 pregnant women with microcephaly to determine whether they were eligible for inclusion in our study. Among them, 66 couples refused genetic testing (karyotype or CMA), six couples were transferred to other hospitals, 12 cases were missing paternal DNA samples, and 16 samples were of insufficient quality to be analyzed. The mean maternal age (on reporting) was 32.4 years (range 22.0–37.3 years), and the median gestational age at the time of microcephaly diagnosis was 30.21 weeks (range 23.00–37.43 weeks). The clinical details of the study cohort are summarized in Table 1.

**TABLE 1:** The clinical details of 224 fetuses with microcephaly in the study.

Maternal age (median)	32.4 (range 22–37.3) years
Gestational weeks (median)^1^	30.21 (range 23–37.43) weeks
Sex of fetuses (M/F)	79/50
Sample types	
Amniotic fluid	149
Cord blood	75
Malformation classification	
Primary microcephaly	38
Syndromic microcephaly	186
Follow-ups^2^	224

^1^ Means the gestational age at microcephaly diagnosis.

^2^ Successful follow-up cases (range 2.1–9.4 years).

### 3.2 Results of karyotype and CMA analysis in prenatal fetal microcephaly cases

Sufficient samples for successful karyotype analysis were obtained from 324 fetuses (224 cases from amniocentesis, 23 cases from chorionic villus sampling, and 77 cases from cordocentesis). Overall, 11.1% (36/324) of fetuses had an abnormal karyotype. 67.9% showed aneuploidy, and Trisomy 21 (5.1%) was the most common abnormality.

CMA identified clinically significant variants in 7 out of 187 cases (3.74%). There was no statistically significant difference in the yield for the syndromic microcephaly group and the primary microcephaly group (*p* = 0.577). Based on the results of CMA, the pCNVs included 17p13.3 microdeletion, 17q12 microdeletion, Xp22.31 microdeletion, and 7q36.3 microduplication (Table 2). For six patients with P/LP CNVs or VUS, the CMA results of the parents were available; one couple refused parental CMA testing. In these six cases, the CNVs identified by CMA in patients 1, 2, 4, 5, and 6 were *de novo*. The CNV detected in patient 7 was inherited from his father, but his father didn’t show the relevant phenotype. This result has been confirmed by ES. The clinical and chromosomal features of seven pCNVs are shown in Table 2.

**TABLE 2:** Pathogenic copy number variation and LP identified by CMA in prenatal fetal microcephaly cases.

Patient	MA	GA at the suspicion of MIC	Associated anomaly	Microarray result	Length	Type	Classification	Outcomes	Parental study
1	33.1	32.4	VSD, Pulmonary atresia	arr[hg19]17p13.3p13.2(525–5874,254)×1	5.87 Mb	Deletion	P	TOP	*de novo*
2	34.25	27.1	—	arr[hg19]21q11.2q22.3(15,006,457–48,097,372)×3	33.09 Mb	Duplication	P	TOP	*de novo*
3	28.2	29.7	Macrogyria	arr[hg19]17p13.3p13.2(526–3522,432)	3.52 Mb	Deletion	P	TOP	NA
4	29.07	26.5	Cerebellar vermis missing	arr[hg19]13q11q34(19,436,286–115,107,733)×3	95.67 Mb	Duplication	P	TOP	*de novo*
5	25.76	28.2	Callosal dysplasia	arr[hg19]17q12(34,822,465–36,378,678)×3	1.56 Mb	Duplication	LP	TOP	*de novo*
6	24.31	33.21	—	arr[hg19]Xp22.31(6885115–7775,073)×0	890 Kb	Deletion	P	TOP	*de novo*
7	27.59	29.71	VSD	arr[hg19]7q36.3(158,136,163–159,119,707)×1	7.87 Mb	Duplication	P	TOP	Paternally inherited

MA, maternal age; GA, gestational age; VSD, ventricular septal defect; P, pathogenic; LP, likely pathogenic; TOP, termination of pregnancy.

### 3.3 Diagnostic variants detected by ES in prenatal fetal microcephaly cases

The 224 cases were divided into two groups: with or without other structural abnormalities detected by ultrasound, known as the primary microcephaly group (38 cases) and the syndromic microcephaly group (186 cases). Among structural abnormalities associated with fetuses suffering from microcephaly, central nervous system defect is the most common. A genetic analysis by ES was performed on 162 pregnant women diagnosed with fetal microcephaly via ultrasound examinations who had negative karyotype and CMA test results. The results of the ES showed that 31 diagnostic genetic variants were identified in 25 genes from 37 fetuses including 19 fetuses (61.29%) that had a *de novo* variant. There were nine (29.03%) cases of autosomal dominant (AD) inheritance, 13 (41.94%) cases of autosomal recessive (AR) inheritance, three (9.68%) cases of X-linked dominant (XLD) inheritance, and two (6.45%) cases of X-linked recessive (XLR) inheritance (These 4 cases of gene deletions and microdeletions were not listed). *De novo* variants included a frameshift variant, two splice variants, 10 missense variants, and three nonsense variants. 12 fetuses (38.71%) inherited the relevant variants (Table 3). The difference in diagnostic rate between primary microcephaly and syndromic microcephaly was significantly different (36.67%, 11/30% vs 15.15%, 20/132; *p* = 0.007). The detection rate of clinically significant variants was significantly higher in the microcephaly group at >34 weeks of gestation than at <34 weeks (28.36%, 19/67% vs 12.63%, 12/95; *p* = 0.012). VUS were found in 33/162 (20.3%) fetuses with microcephaly.

**TABLE 3:** The summary of clinical phenotype and diagnostic variants detected by ES in microcephaly cases.

ID	Patient ID	The clinical phenotype	Gene	Transcripts	Variant	Origin	Inheritance	ACMG classification	Zygosity	Associated condition	Function/pathway	Cerebral MRI finding	References for variants
1	P9445	moderate speech delay, cryptorchidism	KNL1	NM_144508.5	c.5184dup (p.Ile1729fs)	*De novo*	AD	P	Heterozygous	Microcephaly 4, primary, autosomal recessive (MIM: 604321)	Centrosome, spindle and microtubule organization	Enlargeme-nt in the left ventricle	Saadi et al. (2016)
2	P7900	VSD, hyperopia, astigmatism, Mild global DD	ANKRD11	NM_013 275.5	c.3223G>A (p. E1075K)	*De novo*	AD	P	Heterozygous	KBG syndrome (MIM: 148050)	Transcriptional regulation	No abnormality	_
3	A34241	Moderate global DD, Seizure, spastic hemiparesis	HDAC8	NM_018486.3	c.490C>T (p. Arg164Ter)	*De novo*	XLD	P	Heterozygous	Cornelia de Lange syndrome 5(MIM: 300882)	transcriptional regulation	Arachnoidal cyst	Kaiser et al. (2014)
4	P9483	Pulmonary atresia, patent ductus arteriosus, mild feeding difficulties	MYO18B	NM_032608.7	c.4087C>T (p. Arg1363Ter)	Maternal inherited	AR	LP	heterozygous	Klippel-Feil syndrome 4, autosomal recessive, with myopathy and facial dysmorphism (MIM: 616549)	neuronal proliferation and migration	Abnormal gyrus, corpus callosum dysgenesis, everted hippocampi	Alazami et al. (2015)
					c.3639G>A (p. Gly1213 = )	Paternal inherited		VUS	heterozygous				
5	P9125	Blind, Retinal detachment, moderate global DD, vesicoureteral reflux	TUBGCP6	NM_020461.3	c.2066–6A>G	Maternal inherited	AR	LP	Heterozygous	Microcephaly and chorioretinopathy, autosomal recessive, 1 (MIM:251270)	transcriptional regulation	No abnormality	-
					c.4485–31_4485-22delGCCCGCCCTG	Paternal inherited		VUS	Heterozygous	-	-	-	-
6	A33033	Severe global DD, decreased reflexes, severe failure to thrive, congenital cataract, hepatomegaly	OSGEP	NM_017807.4	c.974G>A (p. Arg325Gln)	Paternal inherited	AR	LP	Heterozygous	Galloway-Mowat syndrome (MIM: 617729)	cell cycle regulation	Hypoplasia of cerebellar vermis	Chen et al. (2007)
					c.740G>A (p. Arg247Gln)	Maternal inherited		VUS	Heterozygous				
7	A32966	Mild global DD, mild ID, hypotonia, failure to thrive, short stature, mild truncal adiposity	SLC26A4	NM_000441.2	c.1174A>T (p. Asn392Tyr)	Maternal inherited	AR	P	Heterozygous	Deafness, autosomal recessive 4, with enlarged vestibular aqueduct (MIM:600791)/Pendred syndrome (MIM: 274600)	Regulation of apoptosis and division	Cerebellar hypoplasia	_
					c.919–2A>G	Paternal inherited		P	Heterozygous				
8	A32860	Moderate speech delay, Mild global DD, EEG abnormalities, growth deficiency	SKIV2L	NM_006929.4	c.1120C>T (p. Arg374Ter)	Maternal + Paternal inherited	AR	P	homozygous	Trichohepatoenteric syndrome 2 (MIM:614602)	Centrosome, spindle and microtubule organization	No abnormality	_
9	A32741	Moderate global DD, severe progressive optic atrophy, moderate hyperopia, bilateral hearing loss	COL1A2	NM_000089.3	c.1072G>A (p. Gly358Ser)	*De novo*	AD	P	Heterozygous	Ehlers-Danlos syndrome, arthrochalasia type, 2(MIM:617821)	spindle and microtubule organization	Agenesis of corpus callosum	_
10	P9424	Distal spasticity, neurogenic clubfeet, growth deficiency, hemivertebra	EBP	NM_006579.2	c.328C>T (p. Arg110Ter)	*De novo*	XLD	P	Heterozygous	MEND syndrome (MIM:300960)	Stability of centriole	hypoplasia of the corpus callosum	_
11	A32706	Mild global DD, generalized hypotonia, VSD, torticollis	DOCK6	NM_020812.3	c.807-1G>A	Paternal inherited	AR	LP	Heterozygous	Adams-Oliver syndrome 2 (MIM:614219)	spindle and microtubule organization	cortical malformations	_
					c.377 + 5G>A	Maternal inherited		VUS	Heterozygous				
12	A32409	Moderate global DD, 2 episodes of seizure, inappropriate laughter, PA, muscular hypotonia	ZEB2	NM_014795.3	c.436G>T (p. Glu146Ter)	*De novo*	AD	P	heterozygous	Mowat-Wilsonsyndrome (MIM:235730)	Transcriptional inhibitor	The development or hypoplasia of the corpus callosum	Wakamatsu et al. (2001)
13	A32706	Mild–moderate motor DD, VSD, mild speech delay, short stature, behavior abnormalities, recurrent infections	TRIO	NM_007118.3	c.5302C>T (p. Arg1768Trp)	*De novo*	AD	LP	heterozygous	Intellectual developmental disorder, autosomal dominant 44, with microcephaly (MIM:617061)	cell cycle regulation	No abnormality	Mercer et al. (2008)
14	A31618	Moderate–severe global DD, pulmonary stenosis, muscular hypotonia, VSD	NIPBL	NM_133433.3	c.6983C>A (p. Thr2328Lys)	*De novo*	AD	P	heterozygous	Cornelia de Lange syndrome 1 (MIM:122470)	cell cycle regulation	No abnormality	Braddock et al. (1993)
15	A31843	Moderate–severe global DD, moderate–severe ID, VSD, Hemivertebra, duplication of kidney and hydronephrosis	FANCI	NM_001113378.1	c.3042C>G (p. Cys1014Trp)	Paternal inherited	AR	P	homozygous	Fanconi anemia, complementation group IMIM: 609053)	spindle	Megagyrus, widened lateral ventricle	Savage et al. (2015)
16	A31314	Severe global DD, severe ID, intermittent hyperventilation, distal hypertonia, short stature	PDHA1	NM_000284.3	c.1142_1145dupATCA (p. Trp383SerfsTer6)	*De novo*	XLD	P	Heterozygous	Pyruvate dehydrogenase E1-alpha deficiency (MIM:312170)	cell cycle regulation	schizencephaly	_
17	A31627	Severe global DD, sterotypic movements, tonus dysregulation, feeding difficulties	NIPBL	NM_133433.3	c.6983C>A (p. Thr2328Lys)	*De novo*	AD	LP	Heterozygous	Cornelia de Lange syndrome 1 (MIM:122470)	cell cycle regulation	Enlargeme-nt in the left ventricle	Braddock et al. (1993)
18	P9017	DD, VSD, muscular hypotonia	CLCN5	NM_001127898.3	c.934-1G>T	Maternal inherited	XLR	LP	hemizygous	Hypophosphatemic rickets (MIM:300554)	chromosome condensation, cell cycle regulation	Atrophy of the white matter	_
19	A34124	Learning disability, myopia, short stature, ADHD, short stature	13q14.2-q32.1	chr13:49833482–96743823: 46.9 Mb deletion		*De novo*	-	P	-	-	-	-	-
20	A33056	Fine motor problems, VSD, moderate–severe speech delay, PA	NIPBL	NM_133433	complete gene deletion	-		P	-	Cornelia de Lange syndrome 1 (MIM:122470)	cell cycle regulation	expansion of the lateral ventricle	-
21	P7789	Severe global DD, pain insensitivity, inappropriate laughter, cyanotic seizures	UBE3A	NM_000462	c.2576_2577delAA (p. K859RfsX24)	*De novo*	AD	P	Heterozygous	Angelman syndrome (MIM:105830)	Transcription	Macrogyria	_
22	A33126	Severe global DD, hypotonia, seizures, mild speech delay	CASK	NM_003688.3	c.1889_1895delTCATCCC (p. Leu630ProfsTer51)	*De novo*	XLD	P	Heterozygous	Intellectual developmental disorder and microcephaly with pontine and cerebellar hypoplasia (MIM:300749)	cell cycle regulation	Hypomyelination, thin corpus callosum	Moog et al. (2011)
23	P3376	Moderate global DD, VSD, recurrent subfebrile temperature, short stature, joint laxity	PHC1	NM_004426.3	c.100C>T (p. Arg34Ter)	*De novo*	AR	P	Heterozygous	Microcephaly 11, primary, autosomal recessive (MIM: 615414)	cell cycle regulation	Mild cortical atrophy	Awad et al. (2013)
					c.2041 + 3_2041+6delGAGT	Maternal inherited		VUS	Heterozygous				
24	A33057	Mild–moderate DD, strabismus, short stature, joint laxity, vesicoureteral reflux due to renal tubular ectasia, VSD, chronic obstipation, muscular hypotonia	TUBB2A	NM_001069.2	c.1162A>G (p. Met388Val)	*De novo*	AD	LP	Heterozygous	Cortical dysplasia, complex, with other brain malformations 5(MIM:615763)	microtubule organization	Hypoplasia of cerebellar vermis, thin corpus callosum	Cushion et al. (2014)
25	A35607	Mild–moderate motor DD, short stature	7q11.23	1.44 Mb deletion		*De novo*	_	_	_	Williams-Beuren syndrome (MIM:194050)	_	_	_
26	A33021	Mild–moderate global DD, cyanotic seizures, tonus dysregulation, short stature	WDR62	NM_001083961.1	c.321dupT (p. Asn108Ter)	Paternal inherited	AR	LP	homozygous	Microcephaly 2, primary, autosomal recessive, with or without cortical malformations (MIM: 604317)	neuronal proliferation and migration	callosum dysgenesis, everted hippocampi	Darvish et al. (2010)
27	A35078	Fine motor problems, moderate ID, dysmetria, moderate–severe speech delay, VSD, short stature	PEX1	NM_000466.3	c.2966T>C (p. Ile989Thr)	Paternal inherited	AR	LP	Heterozygous	Peroxisome biogenesis disorder 1A (Zellweger) (MIM: 214100)	Transcription	Absence of corpus callosum	_
					c.1285G>A (p. Val429Ile)	Maternal inherited		VUS	Heterozygous				
28	A34098	Moderate global DD, PA, short stature	LAGE3	NM_006014.5	c.184A>G (p. Ile62Val)	Maternal inherited	XLR	LP	hemizygous	Galloway-Mowat syndrome 2, X-linked (MIM: 301006)	transcription	Macrogyria	Braun et al. (2017)
29	A31066	Moderate global DD, short stature	17p13.3	3.51 Mb deletion		*De novo*	_	_	_	Miller-Dieker lissencephaly syndrome (MIM:247200)	_	_	Schinzel et al. (1988)
30	A32068	Fine motor problems, Mild speech delay, short stature, right plagiocephaly	FANCI	NM_001113378.1	c.3187–2A>G	*De novo*	AR	LP	heterozygous	Fanconi anemia, complementation group I (MIM: 609053)	spindle	Enlarged subarachnoidal space	Savage et al. (2015)
					c.3015G>C (p. Gln1005His)	Paternal inherited		VUS	heterozygous				
31	P9989	Moderate–severe global DD, moderate ID, Dysmetria, short stature, bilateral cryptorchidism	PPFIBP1	NM_003622.4	c.403C>T (p. Arg135Ter)	*De novo*	AR	P	homozygous	Neurodevelopmental disorder with seizures, microcephaly, and brain abnormalities (MIM:620024)	Cell cycle regulation	dysplasia of the corpus callosum	Rosenhahn et al., 2015

XLR, X-linked recessive; P, pathogenic; AD, autosomal dominant; LP, likely pathogenic; AR, autosomal recessive; VUS, variants of unknown significance; XLD, X-linked dominant.

ADHD, attention deficit–hyperactivity disorder; DD, developmental delay; EEG, electroencephalogram; ID, intellectual disability; VSD, ventricular septal defect; PA, pulmonary atresia.

### 3.4 Pregnancy outcomes of the participants

The pregnancy outcomes included 91 terminations of pregnancy (TOP), four cases of intrauterine death, and 129 live births, including 115 full-term births and 14 preterm births. The perinatal outcomes of our cohort are summarized in Table 4. Six fetuses that didn’t receive a genetic diagnosis through ES didn’t detect abnormalities after delivery. Of the 29 fetuses with ES diagnosis of P/LP genetic variants, postmortem or postnatal follow-up was consistent with prenatal diagnosis. However, two fetuses were diagnosed with syndromic microcephaly based on prenatal ultrasound examinations and found to have single-foot varus and hemivertebra, respectively, after birth. Compared to the primary microcephaly group, the syndromic microcephaly group had a higher live birth rate (62.9% (117/186) vs 31.56% (12/38); *p* = 0.000). Of the successfully delivered 129 fetuses, 10 had pathogenic variants detected by ES, and 119 live births were negative for both CMA and ES. Seven patients with pCNVs detected by CMA chose to terminate the pregnancy. Of the 10 live births with pathogenic variants detected by ES, two had variants inherited from the mother and one from the father, and the remaining seven were *de novo* variants. Of the 131 fetuses with negative ES test results, 19 were in the primary fetal microcephaly group, and 112 were in the syndromic microcephaly group. The fetal prognosis was better in the syndromic microcephaly group than in the primary microcephaly group. Fetuses with pathogenic variants detected by ES had a poor prognosis with varying degrees of intellectual disability. In contrast, those with negative results for the CMA and ES tests had a better prognosis. Poor prognoses were recorded, including fetal intrauterine death, neonatal death, severe developmental delay, severe disability, moderate to severe intellectual disability, etc.

**TABLE 4:** The perinatal outcomes in fetuses with microcephaly.

		Syndromic microcephaly vs. primary microcephaly			ES testing results			
Perinatal outcomes	Total (n = 224)	Primary microcephaly (n = 38)	Syndromic microcephaly (n = 186)	*p*-value	CSVs (n = 31)	VUS (n = 30)	Negative (n = 101)	*p*-value
TOP	91(40.63%)	25(65.78%)	66(35.48%)	0.000	21(67.74%)	15(50.00%)	31(30.69%)	0.001
Live birth	129(57.60%)	12(31.56%)	117(62.90%)	0.000	10(32.26%)	14(46.67%)	67(66.34%)	0.001
Intrauterine death	4(1.78%)	1(5.26%)	3(1.61%)	0.520	0	1(3.33%)	3(2.97%)	0.652

CSVs, clinically significant variants; VUS, variants of unknown significance; TOP, termination of pregnancy.

### 3.5 Association between microcephaly, intellectual disability, and abnormal cerebral magnetic resonance images

We found that the severity of intellectual disability (*p* < 0.01, Spearman’s rank correlation) and abnormal cerebral magnetic resonance image (MRI) (*p* < 0.01, Fisher’s exact test) were significantly associated with the severity of microcephaly Growth retardation was observed in 31.01% (40/129) of living infants, mild intellectual disability in 58.14% (75/129), moderate-to-severe intellectual disability in 13.95% (18/129), epilepsy in 26.36% (34/129), and visual impairment in 17.83% (23/129) of the cases. Of the patients who underwent intelligence assessment, the median age at the last assessment was 4.1 years (range: 2.1–9.4). Among 93 patients with intellectual disability, the median score of intelligence was 34 (range: 4–66), and the median score of speech was 35 (range: 21–75). We also found that 82.14% (184/224) of the fetuses with microcephaly were short or had lower height, which suggested that perhaps overall growth retardation/restriction had occurred, leading to both microcephaly and short stature. The mean follow-up time was 4.1 years (range 2.1–9.4 years) for all live births.

## 4 Discussion

In recent years, due to the rapid development of CMA and ES and their application in prenatal diagnosis, the diagnosis rate of CMA has increased by 6%–10% (Wapner et al., 2012; Qi et al., 2020). The utilization of ES increased the rate of genetic diagnosis for fetuses with structural abnormalities by about 10% (Petrovski et al., 2019). Microcephaly is a common clinical phenotype in patients with neurodevelopmental problems. Some studies (Rump et al., 2016; Boonsawat et al., 2019; Shaheen et al., 2019; Lee et al., 2021) investigated the genetic causes in the microcephaly cohort, all participants had postpartum microcephaly. We investigated prenatal microcephaly. In this study, we comprehensively evaluated the genetic causes of 224 cases of prenatal microcephaly using CMA and ES techniques for genetic testing. We discussed their pregnancy outcomes and prognostic follow-ups.

We found a positive diagnosis rate of 3.74% (7/187) for CMA, which was slightly lower than that reported in other studies (von der Hagen et al., 2014) (14.94%). We also recorded a positive diagnosis rate of 19.14% (31/162) for ES, which was higher than CMA and the average diagnosis rate of ES reported in other studies (10%) (Petrovski et al., 2019; Al-Kouatly et al., 2021; Sun et al., 2022). The rate was also slightly higher than that of the previous largest sample size of the microcephaly study, which recorded a positive diagnosis rate of 18.83% (von der Hagen et al., 2014). In this study, the detection rate of CMA was not significantly different between the syndromic microcephaly and primary microcephaly groups. Pathogenic or likely pathogenic variants were detected in participants with microcephaly (19.14%, 31/162), indicating a high additional diagnostic rate of ES for prenatal molecular diagnosis. The difference in the detection rate of ES between the primary microcephaly group and the syndromic microcephaly group was significant.

In the syndromic microcephaly group, the most common combined structural malformations were located in the central nervous system and cardiovascular system, which was similar to the findings of previous studies (Lee et al., 2021). Of these combined malformations of the central nervous system, the most common type was abnormal brain white matter, followed by callosal dysplasia and cortical abnormalities. Among the 31 detected variants, all genes showed varying degrees of involvement in structural brain development abnormalities, except for the small fetal HC. However, transabdominal 2D ultrasound can only display part of the cerebral sulcus. Ultrasound assessment of the cerebral sulcus and gyrus delay should be done with caution and combined with dynamic observations and, if necessary, MRI for diagnosis. MRI is a more sensitive imaging method for identifying abnormalities associated with neurological lesions and microcephaly (Griffiths et al., 2019). Additionally, microcephaly can be associated with other systemic abnormalities, such as cardiac, skeletal system, and cleft lip and palate. In our study, the most common structural abnormality of the heart was a ventricular septal defect. Therefore, we suggest that ultrasound monitoring should be strengthened when detecting fetal microcephaly, especially for the brain structure, such as the sulcus and gyrus and fetal heart structure. As shown in previous studies, we also found here that many fetuses with microcephaly face the risk of intellectual disability. Therefore, we suggest that continuous ultrasound examinations of the fetus with microcephaly should be conducted during pregnancy to evaluate the progress of fetal head circumference.

Also, 61.29% (19/31) of the ES-positive cases were novel variants, which occurred probably because all ES in this study used trio vs. singleton testing. This yielded a higher positive rate and helped in better analyzing novel variants and compound heterozygous variants. Additionally, the participants in other studies were all patients with postnatal microcephaly, while those in our cohort were all patients with prenatal microcephaly. This might have resulted in a low detection rate of genetic variants in prenatal microcephaly due to various reasons (for example, there may have been challenges determining if fetuses have microcephaly). However, the genetic diagnosis rate of postnatal microcephaly in our hospital was 50.13%, which was higher than that reported in previous studies (Rump et al., 2016; Boonsawat et al., 2019; Shaheen et al., 2019; Lee et al., 2021). The genetic diagnosis rate of prenatal microcephaly in our study was significantly different from that of postpartum microcephaly (19.14% vs 50.13%, *p* = 0.000). We suspect that the reason for this difference may be that a proportion of the children born after prenatal diagnosis of microcephaly thought not to need genetic testing after all or some other differences in genetic testing perhaps. This finding indicated if prenatal fetal microcephaly is suspected, a prenatal diagnosis should be conducted immediately to minimize adverse pregnancy outcomes.

In our study cohort, among the 31 pathogenic variants identified by ES, nine (29.03%) were AD, and 13 (41.94%) were AR genetic models. This finding matched the genetics of microcephaly described in previous studies (Rump et al., 2016).In this study, we not only confirmed the previous clinical features related to microcephaly, including intellectual disability, abnormal brain MRI, epilepsy, and short stature, but we also found that some patients had motor disorders, behavioral problems, retinal detachment, and hearing impairment. Our findings confirmed that the severity of microcephaly, fetal growth, and the development performance of microcephaly were not correlated (*p* > 0.05) (Bolduc and Shevell, 2005; Baxter et al., 2009). Furthermore, our study found that variants in genes related to microcephaly are sporadic and involve a large number of genes, which further indicates that the gene variant spectrum of microcephaly is relatively broad.

In the study, we found genetic abnormalities before the birth of a microcephaly fetus. However, because the fetus lacked a unique phenotype, these monogenic diseases were difficult to detect before delivery. Microcephaly usually occurs in the third trimester of pregnancy, which is easy to lose the significance of prenatal diagnosis, which brings great challenges to prenatal diagnosis. We suggested that performing ES and CMA should be used to determine the genetic causes of microcephaly, expand the prenatal phenotype associated with microcephaly genes, and help in distinguishing microcephaly from fetal intrauterine growth restriction (FGR). After adequate genetic counseling, pregnant women can make more accurate pregnancy-related decisions.

Our study still has several limitations. First, in this study, we did not perform a detailed functional assessment of the discovered VUS. Although not classified as P or LP, some VUSs found in this study might help determine the genetic causes of microcephaly and confirm the functions of these genes in future studies. Second, CNVs that did not cover the genomic region on the platform could not be detected. Third, Due to the limitation of chip design, it is impossible to detect all related site abnormalities related to specific syndromes, and negative CMA results cannot completely exclude chromosomal diseases. Fourth, When ES captures the target area, there are phenomena such as uneven capture and capture deviation. Five, due to the relatively expensive price of ES, some couples have abandoned ES testing, leading to the possibility that we may miss some clinically significant variants. Additionally, the long-term prognosis remains unknown since the oldest patient at follow-up was only 9 years old. Moreover, variation analysis and interpretation are still challenging. A prenatal analysis is usually more limited compared to a postnatal analysis, including an often unclear or incomplete interpretation of the phenotype of the fetus. Depending on the gestational age, the phenotype might be incomplete or at least incompletely detectable because abnormalities might occur in late pregnancy (Drury et al., 2015).

To summarize, this was a study in which CMA and ES were performed for the genetic analysis of fetal microcephaly cases. Our findings suggested that CMA and ES tests had a high diagnostic rate for the genetic causes of fetal microcephaly. In this study, we identified 19 novel variants, which expanded the disease spectrum of microcephaly-related genes. Especially, in patients carrying fetuses with syndromic microcephaly and with a negative genetic test result, a definitive genetic diagnosis and a good prognosis might increase confidence in continuing the pregnancy.

## Data Availability

The datasets presented in this study can be found in online repositories. The names of the repository/repositories and accession number(s) can be found in the article/Supplementary Material.
