# Supplementary material for: Genetic diagnosis of fetal microcephaly at a single tertiary center in China
Source: Front Genet. 2023 May 9;14:1112153. doi: 10.3389/fgene.2023.1112153 (PMC10203430; doi:10.3389/fgene.2023.1112153)
Supplement: Supplementary file 1 [file DataSheet1.DOCX]

**Additional file 1. Analysis and interpretation process of fetal ES data**

**Whole exome sequencing (WES)**

Fetal DNA was extracted from chorionic villi, amniocytes, or cord blood according to the gestational age, and DNA from parents and other relatives was obtained from peripheral blood. Exome capture was performed using Agilent SureSelect human exome capture arrays (V5 or V6, Life Technologies), or the xGen Exome Research Panel v1.0 or v2.0 (Integrated DNA Technologies), and the resultant libraries were sequenced on the Illumina HiSeq 2500, HiSeq Xten or Nova Seq 6000 platform with pair-end 150 base pairs (bp) model following the manufacturer’s instructions.

Raw fastq reads were filtered by using Trimmomatic[1] (v0.36) or fastp [2] (v0.20/v0.23) to remove low quality and adapter contaminated reads, leaving clean reads aligned to the human reference genome (GRCh37/Hg19) with BWA [3] (v0.7.17) mem algorithm, with Samtools [4] (v1.3.1/v1.9) and Picard (v2.17.1) converted to BAM format and PCR duplicates were discarded. Genome Analysis ToolKit [5] (GATK v3.6/v3.8) was employed for local indel realignment, base quality recalibration and haplotypecaller variant calling. Variant annotation was conducted with Ensembl’s Variant Effect Predictor [6] (VEP v85/v104) and Annovar [7] (v2017Jul17/v2020Apr01). Allele frequency information from 1000 Genome Project (1000G Phase 3 v5a), Genome Aggregation Database (gnomAD r2.1/v2.1.1), Exome Aggregation Consortium (ExAC r0.3.1) and the Exome Sequencing Project (ESP v2) were annotated. Multiple software such as SIFT [8], Polyphen2 [9], MutationTaster [10], MutationAssessor [11], Provean [12], CADD [13] and REVEL [14] were applied for protein function prediction and Human Splicing Finder [15], MaxEntScan [16], NNSplice [17], and GeneSplicer [18] and SpliceAI [19] [PMID: 30661751] were performed to assess potential impacts on splicing. Gene/variants were additionally annotated according to ClinVar, ClinGen, the professional version of the Human Gene Mutation Database (HGMD professional v2018.2 & v2021.2), previously associated diseases (based on Online Mendelian Inheritance in Man and Orphanet), and known functional domain data (according to UniProtKB and Human Protein Reference Database). Besides, the imprinted gene (Geneimprint and MetaImprint database) and genome segmental duplication region (downloaded from the UCSC genome browser) were added to the VCF file annotation. Two aspects were evaluated for gender determination, average depth of specific genes on chromosome Y and the heterozygous variants percentage on chromosome X. Then KING [20] and PLINK [21] were used to confirm the family pedigree relationship.

Quality control for each sample included an average depth of > 100X and > 96% targeted region with at least 20X in the prospective study, while average depth > 60X and 20X on target coverage > 90% cutoffs were used in the retrospective study. Variants with poor quality were discarded if meeting one of the following criteria, (1) with a depth (DP) <5X; (2) alternate allele proportion (AAP) <0.25; (3) mapping quality (MQ) <40; (4) genotype quality (GQ) <50. All the annotated variants, excluding low quality ones were subject to downstream analysis with the in-house script. Variants with a minor allele frequency (MAF) >5% were filtered out except for those in HGMD, ClinVar and ClinGen B1 exception list [22] (BA1). Next, we mainly focused on genomic regions known or likely associated with the disease. Based on VEP functional consequence, potential protein-altering variants (e.g., missense, start loss, stop gain/loss, frameshift, in-frame insertion/deletion, or canonical splice-site) were retained. To aid data interpretation, major indications for pES for each fetus were extracted from clinical notes and converted into the standard Human Phenotype Ontology (HPO) terms.

A genotype-driven short rare variant list was prioritized for each trio with the help of local population data (more than 10,000 individuals including both patients and healthy individuals) (Figure 1), (1) dominant *de novo* variants; (2) recessive homozygous variants (no homozygotes in the gnomAD2.1 and internal healthy controls); (3) recessive compound heterozygous variants; (4) *De novo* X chromosome variants or rare hemizygous variants inherited from mother; (5) known disease-causing alleles (ClinVar 3- or 4- star variants); (6) predicted truncating variants (nonsense, frameshift, canonical splice sites) with extremely low allele frequency (<0.01%). This short gene/variant list was then reviewed for clinical correlation and potentially relevant variants were classified based on the American College of Medical Genetics and Genomics (ACMG) guideline [22] and ClinGen VCEP gene-specific criteria [23-30] (when applicable). In addition, *bona fide* disease-causing variants (unrelated to fetal phenotype) with zygosity consistent with disease mode of inheritance in ACMG SF2.0 and childhood-onset disease genes were categorized as potential secondary or incidental findings.

Next, for unsolved cases without a clear answer to the clinical question, a comprehensive review of all rare variants in genes potentially related to clinical indications for prenatal diagnosis was performed with the aid of HPO matching. A gene was considered associated with the fetal anomalies meeting one of the following conditions, the clinical phenotypes of the disease gene should: (1) match HPO entry of the fetal phenotype; (2) match the superclass based on HPO or clinical synopsis in OMIM database; (3) be reported in previous cases manifesting the same or similar phenotypes of the fetuses.

In both steps, pES results were classified into five tiers: (1) positive diagnostic result: P/LP variants identified in a disease gene that can interpret (partly or fully) the fetal phenotype; (2) inconclusive: variant of unknown significance (VUS) identified in a disease gene which can explain (partly or fully) the fetal phenotype; (3) incidental findings (IFs): P/LP variants identified in childhood-onset disease gene, unrelated to fetal phenotype; (4) secondary findings (SFs): P/LP variants identified in genes unrelated to fetal phenotype, according to ACMG recommended list [23, 24]; (5) candidate genes: variants (primarily *de novo*) predicted to be deleterious and absent in general population, identified in undefined disease genes that have a paralog gene or previously published data to support the association with fetal anomalies, or based on animal model and tissue expression.

The ES report included positive diagnostic and inconclusive results related to primary prenatal indications. Incidental and secondary findings with a childhood-onset disease were also included in the report, based on consensus between laboratory and clinicians. Secondary findings with a late-onset disease were not routinely reported. For the retrospective cohort, the results were reported to the couples as scientific research data postnatally. For the prospective cohort, the results were reported once the test was concluded.

**References**

1. Bolger AM, Lohse M, Usadel B. Trimmomatic: a flexible trimmer for Illumina sequence data. Bioinformatics 2014; 30(15):2114-2120.

2. Chen S, Zhou Y, Chen Y, Gu J. fastp: an ultra-fast all-in-one FASTQ preprocessor. Bioinformatics 2018; 34(17):i884-i890.

3. Li H, Durbin R. Fast and accurate short read alignment with Burrows-Wheeler transform. Bioinformatics 2009; 25(14):1754-1760.

4. Li H, Handsaker B, Wysoker A, Fennell T, Ruan J, Homer N *et al*. The Sequence Alignment/Map format and SAMtools. Bioinformatics 2009; 25(16):2078-2079.

5. McKenna A, Hanna M, Banks E, Sivachenko A, Cibulskis K, Kernytsky A *et al*. The Genome Analysis Toolkit: a MapReduce framework for analyzing next-generation DNA sequencing data. Genome Res 2010; 20(9):1297-1303.

6. McLaren W, Gil L, Hunt SE, Riat HS, Ritchie GR, Thormann A *et al*. The Ensembl Variant Effect Predictor. Genome Biol 2016; 17(1):122.

7. Wang K, Li M, Hakonarson H. ANNOVAR: functional annotation of genetic variants from high-throughput sequencing data. Nucleic Acids Res 2010; 38(16):e164.

8. Sim NL, Kumar P, Hu J, Henikoff S, Schneider G, Ng PC. SIFT web server: predicting effects of amino acid substitutions on proteins. Nucleic Acids Res 2012; 40(Web Server issue):W452-457.

9. Adzhubei IA, Schmidt S, Peshkin L, Ramensky VE, Gerasimova A, Bork P *et al*. A method and server for predicting damaging missense mutations. Nat Methods 2010; 7(4):248-249.

10. Schwarz JM, Cooper DN, Schuelke M, Seelow D. MutationTaster2: mutation prediction for the deep-sequencing age. Nat Methods 2014; 11(4):361-362.

11. Reva B, Antipin Y, Sander C. Predicting the functional impact of protein mutations: application to cancer genomics. Nucleic Acids Res 2011; 39(17):e118.

12. Choi Y, Chan AP. PROVEAN web server: a tool to predict the functional effect of amino acid substitutions and indels. Bioinformatics 2015; 31(16):2745-2747.

13. Kircher M, Witten DM, Jain P, O'Roak BJ, Cooper GM, Shendure J. A general framework for estimating the relative pathogenicity of human genetic variants. Nat Genet 2014; 46(3):310-315.

14. Ioannidis NM, Rothstein JH, Pejaver V, Middha S, McDonnell SK, Baheti S *et al*. REVEL: An Ensemble Method for Predicting the Pathogenicity of Rare Missense Variants. Am J Hum Genet 2016; 99(4):877-885.

15. Desmet FO, Hamroun D, Lalande M, Collod-Beroud G, Claustres M, Beroud C. Human Splicing Finder: an online bioinformatics tool to predict splicing signals. Nucleic Acids Res 2009; 37(9):e67.

16. Yeo G, Burge CB. Maximum entropy modeling of short sequence motifs with applications to RNA splicing signals. J Comput Biol 2004; 11(2-3):377-394.

17. Reese MG, Eeckman FH, Kulp D, Haussler D. Improved splice site detection in Genie. J Comput Biol 1997; 4(3):311-323.

18. Pertea M, Lin X, Salzberg SL. GeneSplicer: a new computational method for splice site prediction. Nucleic Acids Res 2001; 29(5):1185-1190.

19. Jaganathan K, Kyriazopoulou Panagiotopoulou S, McRae JF, Darbandi SF, Knowles D, Li YI *et al*. Predicting Splicing from Primary Sequence with Deep Learning. Cell 2019; 176(3):535-548 e524.

20. Manichaikul A, Mychaleckyj JC, Rich SS, Daly K, Sale M, Chen WM. Robust relationship inference in genome-wide association studies. Bioinformatics 2010; 26(22):2867-2873.

21. Chang CC, Chow CC, Tellier LC, Vattikuti S, Purcell SM, Lee JJ. Second-generation PLINK: rising to the challenge of larger and richer datasets. Gigascience 2015; 4:7.

22. Ghosh R, Harrison SM, Rehm HL, Plon SE, Biesecker LG, ClinGen Sequence Variant Interpretation Working G. Updated recommendation for the benign stand-alone ACMG/AMP criterion. Hum Mutat 2018; 39(11):1525-1530.

23. Miller DT, Lee K, Chung WK, Gordon AS, Herman GE, Klein TE *et al*. ACMG SF v3.0 list for reporting of secondary findings in clinical exome and genome sequencing: a policy statement of the American College of Medical Genetics and Genomics (ACMG). Genet Med 2021; 23(8):1381-1390.

24. Kalia SS, Adelman K, Bale SJ, Chung WK, Eng C, Evans JP *et al*. Recommendations for reporting of secondary findings in clinical exome and genome sequencing, 2016 update (ACMG SF v2.0): a policy statement of the American College of Medical Genetics and Genomics. Genet Med 2017; 19(2):249-255.
